# Arsenite Effects on Mitochondrial Bioenergetics in Human and Mouse Primary Hepatocytes Follow a Nonlinear Dose Response

**DOI:** 10.1155/2017/9251303

**Published:** 2017-01-09

**Authors:** Hemantkumar Chavan, Pamela Christudoss, Kristen Mickey, Robert Tessman, Hong-min Ni, Russell Swerdlow, Partha Krishnamurthy

**Affiliations:** ^1^Department of Pharmacology, Toxicology and Therapeutics, University of Kansas Medical Center, Kansas City, KS 66160, USA; ^2^Department of Clinical Biochemistry, Christian Medical College, Vellore 632004, India; ^3^Department of Anatomy and Cell Biology, University of Kansas Medical Center, Kansas City, KS 66160, USA

## Abstract

Arsenite is a known carcinogen and its exposure has been implicated in a variety of noncarcinogenic health concerns. Increased oxidative stress is thought to be the primary cause of arsenite toxicity and the toxic effect is thought to be linear with detrimental effects reported at all concentrations of arsenite. But the paradigm of linear dose response in arsenite toxicity is shifting. In the present study we demonstrate that arsenite effects on mitochondrial respiration in primary hepatocytes follow a nonlinear dose response. In vitro exposure of primary hepatocytes to an environmentally relevant, moderate level of arsenite results in increased oxidant production that appears to arise from changes in the expression and activity of respiratory Complex I of the mitochondrial proton circuit. In primary hepatocytes the excess oxidant production appears to elicit adaptive responses that promote resistance to oxidative stress and a propensity to increased proliferation. Taken together, these results suggest a nonlinear dose-response characteristic of arsenite with low-dose arsenite promoting adaptive responses in a process known as mitohormesis, with transient increase in ROS levels acting as transducers of arsenite-induced mitohormesis.

## 1. Introduction

Inorganic arsenite is a naturally occurring metalloid that is ubiquitously present in the environment. In the world millions of people are exposed to arsenite through ingestion of contaminated drinking water [1]. Chronic exposure of humans to arsenite and arsenite containing compounds is strongly associated with a myriad of health effects including increased incidence of tumors of the bladder, skin, and lung [2–10]. In addition to its carcinogenic effect, epidemiological studies have also associated chronic arsenite exposure with an increased risk in many human nonmalignant diseases, such as cardiovascular and peripheral vascular disease [11], chronic lung disease [12, 13], developmental anomalies [14, 15], and hematological disorder [16, 17].

The liver has long been identified as a target organ of arsenic exposure [18–20]. Its importance as an organ for arsenic biotransformation is well established and arsenic exposure has been linked to both nonmalignant and malignant hepatic abnormalities [21, 22]. Despite the toxic effect of arsenite on the liver, recent epidemiological studies suggest that exposure to low doses of environmental arsenite may unexpectedly be associated with concentration-related adaptive responses that promote slight growth advantage of transformed cells in culture [23, 24] and reduced DNA damage in the liver of rats [25, 26]. Based on studies in flies and in worms, it has been suggested that such concentration dependent adaptive responses are dependent on mitochondrial ROS [27, 28]. However, whether such mitochondrial ROS also plays a role in the nonlinear responses seen in liver is not clearly defined. Further, whether the growth responses seen with transformed cells are applicable in vivo or to primary cells in culture is not clear.

The purpose of the current study was an attempt to define the broad components of arsenite nonlinear effects in human and mouse primary hepatocytes and explore the role of mitochondria in this process. Primary hepatocytes were chosen over in vivo studies because of the transient, variable, and uncertain nature of tissue exposure in in vivo studies that might be of little value for evaluating dose response for mechanistic studies.

## 2. Materials and Methods

### 2.1. Cell Culture

The human hepatoma cell line HepG2 was purchased from American Type Culture Collection (Manassas, VA). Cells were maintained in Dulbecco's Modified Eagle's Medium supplemented with 10% fetal bovine serum (FBS), 100 units/mL penicillin-streptomycin, and 2 mM L-glutamine at 37°C in a humidified atmosphere of 95% air and 5% CO_2_.

### 2.2. Primary Mouse and Primary Human Hepatocyte Culture

Primary mouse hepatocytes were isolated and cultured as described previously [29].

Primary human hepatocytes were isolated from human tissues obtained with informed consent from each patient, according to the ethical and institutional guidelines approved by the University of Kansas Medical Center Institutional Review Board. All liver specimens were obtained in accordance with a Human Subject Committee approved protocol from patients undergoing a hepatic resection procedure or from donor organs. Primary human hepatocytes from liver specimens were isolated as previously described [30]. Briefly, the liver tissue was perfused with calcium, magnesium, and phenol red free HBSS (HyClone Cat #SH30588.02) containing EGTA (1.0 mM). The perfused liver tissue was then gently chopped with sterile scissors to release hepatocytes. Hepatocytes were isolated from the other cell types in the suspension by low speed centrifugation at 80*g* for 5 min at 4°C. The isolated hepatocytes were plated on collagen coated 24- and 96-well plates (Becton-Dickinson). The plating densities for mouse hepatocytes were 0.5 × 10^6^ and 0.4 × 10^6^ viable cells/well for 24- and 96-well plates, respectively. Human hepatocytes that are smaller in size than mouse hepatocytes were plated in 96-well plates at 0.5 × 10^6^ viable cells per well. The plating media was Williams' Medium E (Life Technologies Cat #A12176-01) supplemented with l-glutamine (2 mM) (Life Technologies Cat #35050-061), HEPES (10 mM), insulin (10^−7^ M), dexamethasone (10^−7^ M), penicillin (100 U/mL), streptomycin (100 *μ*g/mL), and amphotericin B (0.25 *μ*g/mL). The same medium without FBS was used to culture attached hepatocyte monolayers for 2-3 days at 37°C in a 95% air/5% CO_2_ atmosphere. The medium was changed daily.

### 2.3. Drug Treatment

For arsenite treatment, culture medium was replaced with fresh media containing the appropriate concentration of sodium arsenite dissolved in water and incubated at 37°C for 16 hours. All arsenite solutions were made fresh in slightly acidic (pH 6.8) Milli-Q water and immediately stored in dark filled microbottles to preserve the As (III) species [31].

### 2.4. Cytotoxicity Assay

Cell viability was measured in logarithmically growing HepG2 cells as previously described [32]. Cytotoxicity of primary hepatocytes was measured in attached hepatocyte monolayers cultured overnight in Williams' E Medium without FBS. Briefly, overnight cultured hepatocyte monolayers were exposed to increasing concentrations of sodium arsenite for an additional 16 h as described above. At the end of 16 h arsenite exposure, cell viability was determined using 3-[4,5-dimethylthiazol-2-yl]-2,5-diphenyl-tetrazolium bromide. Absorbance at 590 nm was measured with a kinetic microplate reader (BioTek, Winooski, VT) and was used as a measure of cell viability. IC_50_ values were calculated based on absorbance and arsenite concentration using Sigma Plot XII.

### 2.5. Mitochondria Stress Test

Oxygen consumption rate (OCR) was measured in real-time, in an XF24^3^ Extracellular Flux Analyzer (Seahorse Bioscience, Billerica, MA) as per manufacturer guideline. Briefly, HepG2 cells and human or mouse primary hepatocytes were seeded in XF^24^-V7 plates (50,000 cells for HepG2 and 30,000 cells for mouse or human primary hepatocytes per well in 100 *μ*L). After 6 h, 400 *μ*L media with sodium arsenite was added to achieve a final concentration of 0, 0.3, 0.6, 1.25, 2.5, 5, and 10 *μ*M arsenite (*n* = 5 wells). Cells were further incubated overnight at 37°C, 5% CO_2_. The XF24^3^ sensor cartridge was hydrated with 1 mL calibration buffer per well overnight at 37°C. The sensor cartridge was loaded with oligomycin (1 *μ*M, port A), carbonyl cyanide 4-(trifluoromethoxy)phenylhydrazone (FCCP, 0.5 *μ*M for HepG2 and 0.3 *μ*M for mouse or human primary hepatocytes, port B), and rotenone plus antimycin A (1 *μ*M each, port C) to measure the bioenergetics profile. Cells were washed twice with prewarmed XF assay medium containing 25 mM glucose and incubated in XF assay medium at 37°C without CO_2_ for 1 h. In case of mouse and human primary hepatocytes XF assay media was supplemented with 1 mM sodium pyruvate. Once the sensor cartridge was equilibrated, the calibration plate was replaced with the cell plate to measure OCR.

### 2.6. Immunoblot Analysis

For immunoblot analysis, cell lysates were prepared in 1x RIPA buffer (Thermo Scientific, Waltham, MA) with protease inhibitors (Roche Applied Science). Approximately 25 *μ*g of total protein was analyzed by polyacrylamide gel electrophoresis (PAGE). Primary antibodies were used to detect SOD2 (Abcam; Cat #ab13533, Cambridge, MA), catalase (Cell Signaling, Cat #8841, Danvers, MA), GAPDH (Cell Signaling, Cat #2118), actin (Sigma-Aldrich, Cat #A5441, St. Louis, MO), HO-1 (Enzo Life Sciences, Cat #ADI-SPA-895, Farmingdale, NY), and OXPHOS complexes (Abcam, mouse #ab110413, human #ab13533). Immunoreactive proteins were detected using polyclonal goat anti-rabbit or anti-mouse horseradish peroxidase IgG secondary antibodies (Thermo Scientific, Waltham, MA) and visualized using Supersignal™ chemiluminescent horseradish peroxidase substrate (Thermo Scientific, MA). Densitometry analysis was performed using ImageJ analysis software (National Institutes of Health).

### 2.7. Complex I and IV Activity Assay

Complex I and IV activities were measured using the Complex I Enzyme Dipstick Assay kit or Complex IV Dipstick Assay kit (Abcam, Cambridge, UK) as per the manufacturer protocol. Briefly, 50 *μ*g total protein was allowed to wick on a dipstick which allowed Complex I or IV protein to be immunocaptured with anti-human or anti-mouse Complex I or IV antibody. The dipstick was then immersed in Complex I or IV activity buffer solution to form a blue-purple precipitate at the Complex I or IV antibody line on the dipstick. The signal intensity was measured by ImageJ to analyze the activity.

### 2.8. ATP Extraction from Cells

A total of ×10^6^ cells were treated with different concentrations of arsenite for 16 hr. Cells were lysed with 0.5 mL of 0.5 M perchloric acid on ice for 5 min. The extraction mixture was centrifuged at 16,000 ×g for 10 min at 4°C. The supernatant was quickly neutralized to ~pH 6.0 with 4 M potassium bicarbonate. The neutralized supernatant was incubated for 30 min on ice to precipitate potassium perchlorate which was removed by centrifugation at 16,000 ×g for 10 min at 4°C. The supernatant was stored at −80°C until further analysis.

### 2.9. ATP Measurement by HPLC

ATP standards were made in 0.5 M perchloric acid neutralized with potassium bicarbonate as described above. ATP standards were linear for the dilution range used in this study (0.5 *μ*M–1 mM). The HPLC instrument (Shimadzu Instruments Inc., USA) was equipped with an LC-20AD pump system and SPD-M20A diode array detector. Supelcosil™ LC-18 150 × 4.6 mm column (5 *μ*m particle size) was used for separation of ATP. HPLC separation was achieved using gradient elution. Mobile phases A and B consisted of 0.1 M monopotassium phosphate and 0.008 M tetrabutylammonium hydrogen sulfate dissolved in deionized water and adjusted to pH 6.0. Two-percent acetonitrile was added to the mobile phase A while mobile phase B consisted of 30% acetonitrile. The elution program was as follows: 2.5 min 100% A, 0% B; 3.5 min 90% A, 10% B; 1 min 80% A, 20% B; 5 min 60% A, 40% B; 8 min 0% A, 100% B; and 10 min 100% A, 0% B. Flow rate of the mobile phase was 1 mL/min, while the injection volume was 50 *μ*L. The autosampler was set at 4°C and the column was maintained at 30°C. ATP in the samples was identified by comparison with the retention time of the standard (~7 min), while the concentration was determined using a standard curve.

### 2.10. Measurement of Mitochondrial ROS in Primary Hepatocytes

For live cell imaging, primary hepatocytes cultured on cover glass chamber slides were treated with sodium arsenite for 16 h. At the end of treatment, hepatocytes were washed once with phenol red free William's E medium followed by incubation with 500 nM MitoSOX™ Red (Invitrogen) in phenol red free William's E medium for 15 min. Cells were then washed with Hanks Balanced Salt Solution (HBSS) and imaged on a Nikon Ti inverted fluorescence microscope with heated environmental chamber.

For spectrofluorometric quantitation of mitochondrial reactive oxygen species, cells were loaded with MitoSOX Red as for imaging experiments. At the end of MitoSOX Red incubation, cells were washed with HBSS and lysed in lysis buffer (100 mM KCl, 10 mM HEPES, 1.5 mM MgCl_2_, 0.1 mM ethylene glycol tetraacetic acid, 0.5 mM dichlorodiphenyltrichloroethane, and 0.5% NP40). MitoSOX Red fluorescence in the cell lysate was measured at 510/580 (excitation/emission). Acetaminophen (APAP; 5 mM), shown previously to generate mitochondrial oxidative stress, was used as a positive control. The measured fluorescence value was expressed as a fold change compared with that of untreated control.

### 2.11. Determination of Necrosis

After 16 h of arsenite treatment, cells were double-stained with 8 *μ*g/mL Hoechst 33258 and 1 *μ*M SYTOX Green [33]. Hepatocytes were incubated with Hoechst for 10 min. SYTOX was added just before analysis. After staining, the culture dishes were observed under an Olympus fluorescence microscope. Quantitation of total and necrotic cells (SYTOX Green positive) was performed by counting more than 1000 cells in 10 different fields.

### 2.12. Determination of Primary Hepatocyte Proliferation

Proliferation of mouse primary hepatocytes was evaluated using a chemically defined serum-free medium (mouse hepatocyte growth media; MHGM) that allows growth of mouse hepatocytes in primary culture as described [34]. Briefly, mouse hepatocytes were cultured for 48 h prior to arsenite exposure for an additional 16 h. At the end of incubation, the media was replaced with fresh MHGM containing 35 ng/per mL hepatocyte growth factor and cultured for an additional 24 h. Hepatocyte growth was assessed by measuring DNA synthesis following incorporation of BRDU into DNA. BRDU incorporation into DNA was assessed by the use of Invitrogen BRDU staining kit (Life Technologies).

### 2.13. Statistical Analysis

Statistical analysis of the observed values was performed using Student's *t*-test and one-way ANOVA where appropriate. Statistically significant results from ANOVA analysis were computed further using the Tuckey post hoc test. All calculations were performed with SPSS statistical software package (SPSS Inc., Chicago, IL). All values are expressed as either mean ± SD or ±s.e.m.

## 3. Results

### 3.1. Arsenite Toxicity Profile of Primary Hepatocytes and Human Hepatomas

**Figure 1:**
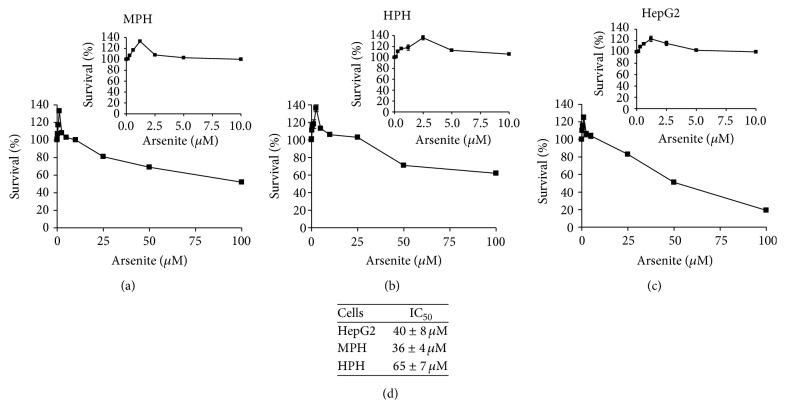
Nonlinear dose response of arsenite in primary hepatocytes. Growth and survival response of (a) mouse primary hepatocytes, (b) human primary hepatocytes, and (c) human hepatoma cell line HepG2 exposed to increasing concentration of sodium arsenite for 16 h. (d) Arsenite IC_50_ values for HepG2, mouse primary hepatocytes, and human primary hepatocytes. Growth and survival were measured using the MTT assay as described in methods. Results are representative of three independent experiments. The insets in (a), (b), and (c) show the toxicity profile at arsenite concentrations from 0 to 10 *μ*M. Values represent mean ± SD. Survival values were significantly different from vehicle treated control cells; *P* < 0.01.

We first evaluated the dose-response relationship of arsenite on primary hepatocyte toxicity. For this purpose human and mouse primary hepatocytes were exposed to increasing concentrations of sodium arsenite (0–100 *μ*M) for 16 hours. We found that at arsenite concentrations of <10 *μ*M primary hepatocytes showed little or no toxicity as measured by the trypan blue exclusion assay (data not shown). In general human primary hepatocytes (HPH) were more resistant to sodium arsenite treatment compared to either mouse primary hepatocytes (MPH) or human hepatoma HepG2 cells (Figure 1(d)). Interestingly, however, we observed that the MTT profile of arsenite in primary hepatocytes followed a nonlinear dose response (Figures 1(a) and 1(b)). At arsenite concentrations of <5 *μ*M primary hepatocytes showed a positive MTT profile. However, at concentrations of >5 *μ*M arsenite appeared to decrease primary hepatocyte MTT profile. These results are consistent with previous reports of MTT profile observed in vitro [35–37].

### 3.2. Arsenite Effect on Mitochondrial Bioenergetics in Primary Hepatocytes

The MTT assay is generally used as a measure of cell proliferation and by analogy cytotoxicity. However, in principle, the assay is a reflection of the metabolic state of the cell. Given that mitochondria play an important role in defining the metabolic state of the cell and that mitochondrial dysfunction has been suggested to play a role in arsenite-induced pathogenesis in liver [38–41], we next evaluated the dose-response relationship of arsenite on mitochondrial bioenergetics. For these studies we chose to use arsenite concentrations between 0 and 10 *μ*M with the rationale that these concentrations would provide a reasonable representation of environmentally relevant, low to moderate arsenite exposure levels. Further we hypothesized that these concentrations would help avoid cellular responses that might be associated with overt cell death that is observed at higher concentrations (Figure 1).

**Figure 2:**
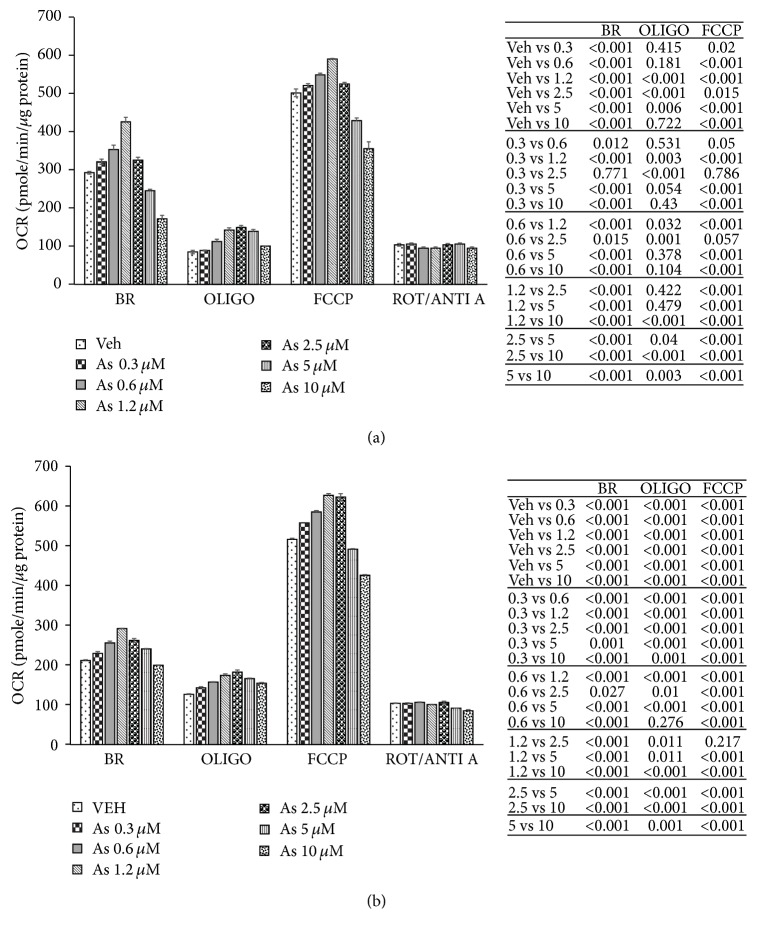
Arsenite effect on mitochondrial bioenergetics in primary hepatocytes. Mitochondrial bioenergetic profile of (a) mouse and (b) human primary hepatocytes in response to increasing concentration of arsenite. Primary hepatocytes in the presence of glucose were exposed sequentially to oligomycin (OLIGO), carbonyl cyanide-p-trifluoromethoxyphenylhydrazone (FCCP), and rotenone/antimycin A (ROT/ANTI A) as described in methods. Nonmitochondrial respiration after the final addition of ROT/ANTI A was subtracted from the other values. BR, basal respiration; OLIGO, oligomycin-sensitive or ATP-linked respiration; FCCP, maximal respiration in the presence of FCCP. Tables to the right of (a) and (b) provide the statistical analysis of the bioenergetic profile. Results representative of 5 independent experiments. Each independent experiment was run using an independent mouse and an independent human liver (representing 5 different mouse and 5 different human livers). Bioenergetic profile data presented as mean ± SD.

Hepatocyte mitochondrial bioenergetics in response to arsenite exposure was evaluated using the Seahorse extracellular flux analyzer, which allows for real-time measurements of oxygen consumption, and provides an overall assessment of the bioenergetic function of the cell. We found that at concentrations of <5 *μ*M arsenite increased mitochondrial oxygen consumption and mitochondrial activity in both mouse (Figure 2(a)) and human (Figure 2(b)) primary hepatocytes. In contrast at concentrations of >5 *μ*M arsenite decreased oxygen consumption and mitochondrial activity in a dose dependent manner in both mouse (Figure 2(a)) and human (Figure 2(b)) hepatocytes. These results suggest that the effects of arsenite on mitochondrial activity in primary hepatocytes might follow a nonlinear dose response.

### 3.3. Arsenite Effect on Mitochondrial Proton Circuit in Primary Hepatocytes

**Figure 3:**
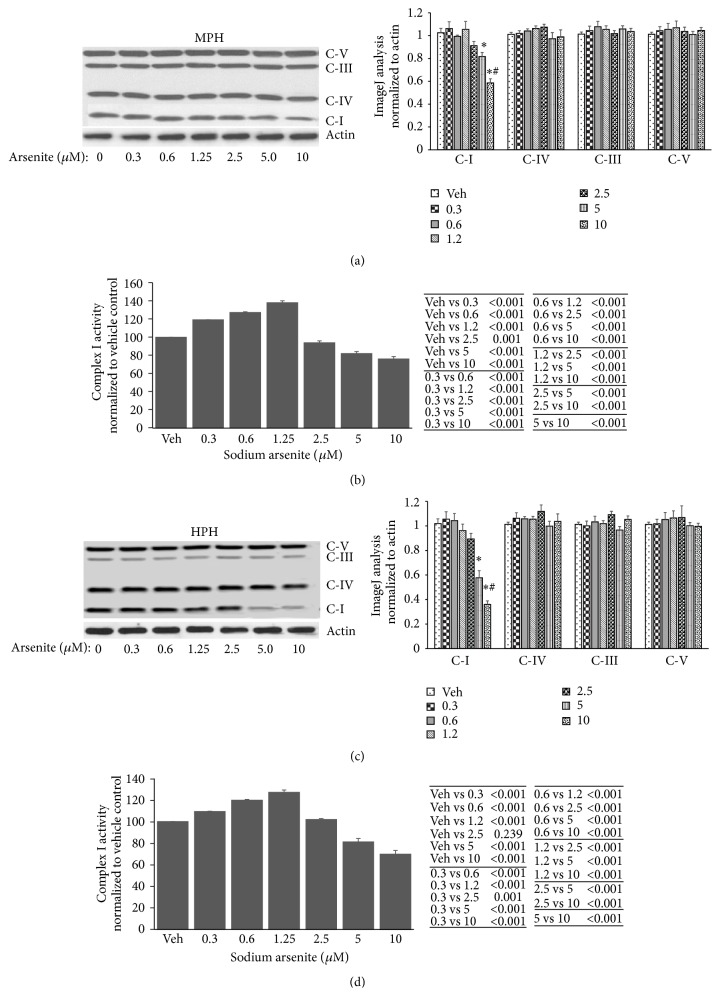
Arsenite effect on mitochondrial respiratory complex expression and activity in primary hepatocytes. ((a) and (c)) Immunoblot analysis of mitochondrial respiratory complex expression in (a) mouse and (c) human primary hepatocytes. Histograms to the right of (a) and (c) show ImageJ analysis of the respective immunoblots. ((b) and (d)) Complex I activity in (b) mouse and (d) human primary hepatocytes. Tables to the right of (b) and (d) provide statistical analysis of Complex I activity data, respectively. Results representative of four independent experiments. Complex I activity data presented as mean ± SD. ImageJ analysis data are normalized to actin and presented as mean ± SD. ^**∗**^Significantly different from vehicle and 0–2.5 *μ*M arsenite treated hepatocytes; *P* < 0.01. ^#^Significantly different from 5 *μ*M arsenite treated hepatocytes; *P* < 0.01. C-I, Complex I; C-IV, Complex IV; C-III, Complex III; C-V, Complex V.

Previous studies have shown that the mitochondrial electron transport chain (ETC) is central to mitochondrial bioenergetics. Thus, in an attempt to define the mechanistic basis for the nonlinear effects of arsenite on mitochondrial bioenergetics, we evaluated arsenite effects on mitochondrial respiratory complex expression and activity. We found that at higher concentrations (>5 *μ*M) arsenite decreased Complex I expression in a dose dependent manner in both mouse (Figure 3(a)) and human (Figure 3(c)) primary hepatocytes. Consistent with decreased Complex I expression, arsenite at high concentrations dose dependently inhibited Complex I activity in both mouse and human primary hepatocytes (Figures 3(b) and 3(d), resp.). In contrast low arsenite concentrations increased Complex I activity in both mouse (Figure 3(b)) and human (Figure 3(d)) primary hepatocytes without any significant change in complex expression (Figures 3(a) and 3(c), resp.). These results suggest that mitochondrial respiratory differences seen with arsenite concentrations of 0–10 *μ*M might result from increased activity of Complex I at low doses (<5 *μ*M) while high dose arsenite (>5 *μ*M) results in decreased ETC expression and function.

### 3.4. Arsenite Effects on Mitochondrial Respiration Results in Proton Leak

The predominant physiological contribution of the proton circuit is the generation of ATP by oxidative phosphorylation. Thus, we tested whether changes to cellular ATP demand in response to arsenite exposure could contribute to the differential respiratory complex activity observed in hepatocytes. However, we found no significant difference in cellular ATP levels in response to arsenite exposure (Supplementary Figure  1 in Supplementary Material available online at https://doi.org/10.1155/2017/9251303).

In the absence of ATP synthesis, the proton circuit is largely completed by the proton leak. Interestingly when oligomycin was added to inhibit oxidative phosphorylation to measure coupling efficiency, primary hepatocytes demonstrated increased leak respiration independent of the arsenite concentration to which they were exposed (Figure 2). This suggests that arsenite causes respiratory uncoupling with increase in proton leak independent of the nonlinear dose response of arsenite on mitochondrial respiration.

### 3.5. Arsenite-Induced ROS Production in Primary Hepatocytes

**Figure 4:**
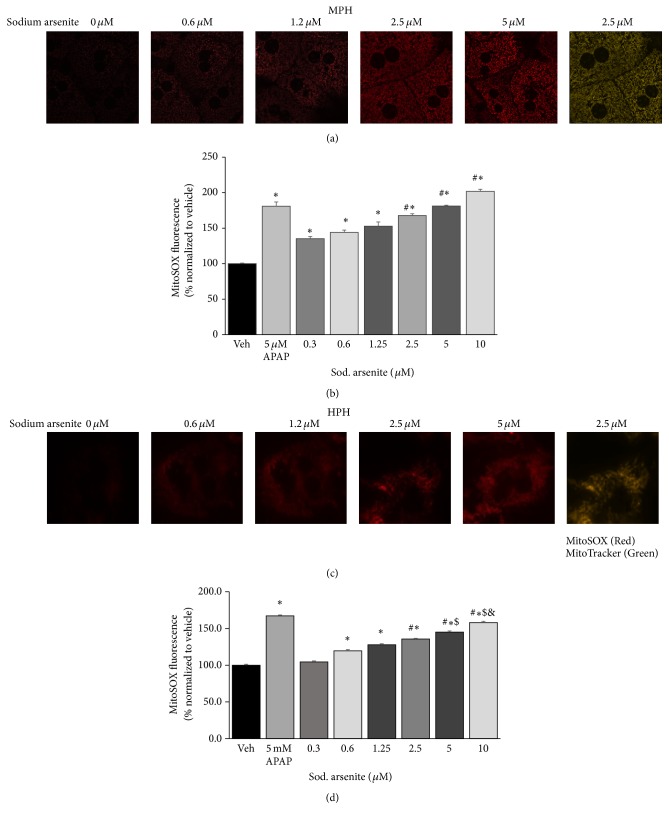
Arsenite induced reactive oxygen radicals (ROS) in hepatocytes. ((a) and (c)) Live cell imaging of MitoSOX fluorescence and ((b) and (d)) spectrofluorometric analysis of mitochondrial reactive oxygen production (MitoSOX fluorescence) in lysates of ((a) and (b)) mouse and ((c) and (d)) human primary hepatocytes. Mitochondrial localization of MitoSOX was confirmed using MitoTracker (mitochondria). Overlay (yellow) represents colocalization of MitoSOX and MitoTracker. Results representative of three independent experiments. ^**∗**^Significantly different from vehicle control cells; *P* < 0.01. ^#^Significantly different from 0.3 and 0.6 *μ*M arsenite treated cells; *P* < 0.05. ^$^Significantly different from 2.5 *μ*M arsenite treated cells; *P* < 0.05. ^&^Significantly different from 5 *μ*M arsenite treated cells; *P* < 0.01. Values in (b) and (d) represent mean ± SD. APAP, acetaminophen.

The mitochondrial leak respiration in the presence of oligomycin is composed of proton leak that may or may not contribute to ROS generation. Thus we used live cell imaging of MitoSOX oxidation to determine if arsenite-induced mitochondrial leak respiration contributed to ROS generation in primary hepatocytes. Consistent with the increased proton leak, arsenite-induced ROS production was linear, with increasing concentration of arsenite promoting increased ROS production in a dose dependent manner in both mouse (Figures 4(a) and 4(b)) and human (Figures 4(c) and 4(d)) primary hepatocytes.

### 3.6. Adaptive Response to Arsenite in Primary Hepatocytes and Hepatomas

**Figure 5:**
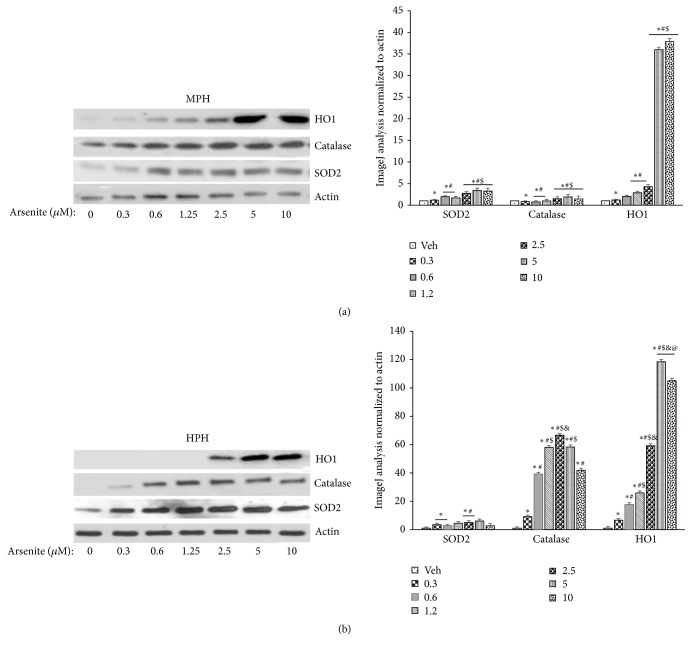
Adaptive response to arsenite in primary hepatocytes. ((a) and (b)) Immunoblot analysis of heme oxygenase (HO1), catalase, and superoxide dismutase 2 (SOD2) expression in (a) mouse and (b) human primary hepatocytes. Histograms to the right of (a) and (b) show ImageJ analysis of the immunoblot data presented in (a) and (b), respectively. Results representative of three independent experiments. ImageJ analysis data are normalized to actin and presented as mean ± SD. ^*∗*^Significantly different from vehicle treated cells; *P* < 0.01. ^#^Significantly different from 0.3 uM arsenite treated cells; *P* < 0.01. ^$^Significantly different from 0.6 and 1.2 uM arsenite treated cells in panel (a); *P* < 0.01. ^&^Significantly different from 1.2 uM arsenite treated cells in panel (b); *P* < 0.01. ^@^Significantly different from 10 uM arsenite treated cell in panel (b); *P* < 0.01.

**Figure 6:**
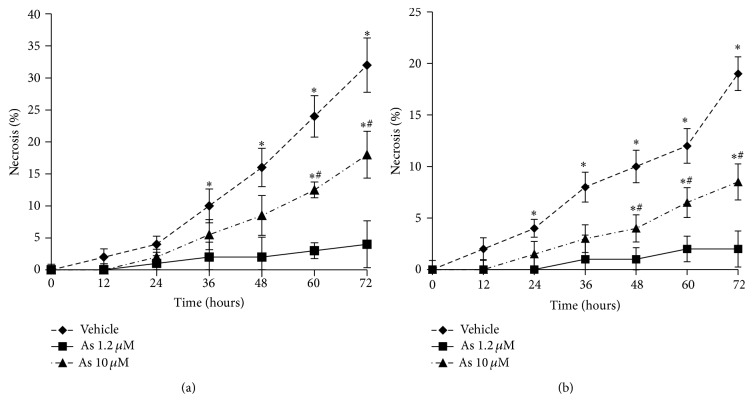
Arsenite induces a primary hepatocyte phenotype that resists oxidant stress. Arsenic and vehicle alone treated (a) mouse and (b) human primary hepatocytes were exposed to a toxic oxidative stress from 100 *μ*M H_2_O_2_. Cells from both groups were exposed to H_2_O_2_ at time = 0 and necrosis was determined using SYTOX Green over 72 h following exposure. Data presented as mean ± SD. ^**∗**^Significantly different from vehicle treated hepatocytes at the designated time point; *P* < 0.01. ^#^Significantly different from 10 uM arsenic treated hepatocytes at the designated time point: *P* < 0.01.

Previous studies in transformed cell lines have suggested reversal in the direction of gene expression change between low and high arsenite concentrations [42]. These changes appear to involve oxidative stress response, proteotoxicity, and proliferative signaling at low doses and predominantly apoptotic genes at high doses [42]. Thus we next tested which, if any, of these changes were impacted by arsenite in primary hepatocytes. We found that arsenite at concentrations as low as 0.3 *μ*M resulted in the activation of the antioxidant stress response which reached a point of saturation beyond arsenite concentration of 2.5 *μ*M (Figure 5). Consistent with this stress response when primary hepatocytes previously exposed to submicromolar concentration of arsenite were challenged with a high concentration of H_2_O_2_, they were significantly more resistant to H_2_O_2_ induced toxicity compared to untreated cells (Figure 6).

**Figure 7:**
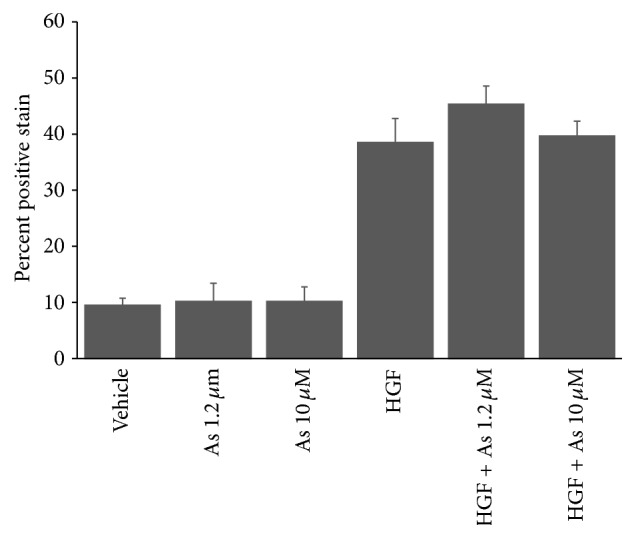
Arsenite effects on mouse hepatocyte proliferation. Mouse hepatocytes exposed to arsenite for 16 h on day 2 of culture were treated with hepatocyte growth factor on day 3. Proliferation in response to arsenite and HGF was evaluated by incorporation of bromodeoxyuridine (BRDU) into DNA. *y*-axis indicates percent of labeled nuclei due to incorporation of BRDU into DNA. Results presented as mean ± SD. Results representative of two independent experiments. HGF positive stain was significantly different from vehicle and arsenic only treated cells: *P* < 0.01.

We next tested if arsenite treatment stimulated proliferation signals in primary hepatocytes. Proliferation of mouse hepatocytes was evaluated as previously described [34]. Although arsenite alone did not produce any significant increase in proliferative signals in mouse primary hepatocytes, pretreatment with arsenite led to a modest increase in the percent of labeled nuclei in response to hepatocyte growth factor, suggesting increased DNA synthesis, an indirect indicator of proliferation. However, this response did not reach statistical significance (Figure 7).

## 4. Discussion

The data presented support the hypothesis that arsenite effects on mitochondrial respiration in primary hepatocytes follow a nonlinear dose response. In vitro exposure of primary hepatocytes to an environmentally relevant, moderate level of arsenite results in increased oxidant production that appears to arise from changes in the expression and activity of respiratory Complex I of the mitochondrial proton circuit. In primary hepatocytes the excess oxidant production appears to elicit adaptive responses that promote resistance to oxidative stress and a propensity to increased proliferation. It is to be noted, however, that the results from our studies are predominantly in vitro and that further in vivo studies are required to confirm these observations.

Historically, mitochondrial ROS production and oxidative damage have been associated with toxicity. However, numerous findings have emerged in recent years indicating that oxidants in general and mitochondrial ROS in particular may act as secondary messengers in many cellular pathways, including those that promote metabolic health and longevity [25, 43, 44]. In our studies, arsenite-induced ROS production in primary hepatocytes resulted in the activation of ROS defense mechanisms that appeared to promote stress resistance. Conceptually these results suggest that low-level stress such as those experienced at low-dose arsenite concentrations may promote adaptive processes that support metabolic health while at high-level stress these processes are unquestionably detrimental.

An increasing body of evidence suggests that the site of ROS production significantly contributes to their apparent dual nature on cellular physiology. In this context mitochondrial respiratory Complex I has been shown to contribute significantly to this dual nature on cellular physiology [27, 45, 46]. In our studies a more detailed examination of the mechanism responsible for the nonlinear mitochondrial bioenergetic response of primary hepatocytes to arsenite also suggests a role for Complex I of the mitochondrial electron transport chain. Although the mechanism by which low-dose arsenite increases Complex I activity is not clear at the present time, the results in general are consistent with experimental manipulations that increase the redox potential of Complex I. However, it is to be noted that the correlation between ROS and overall cellular health might not be a cause-effect relationship but rather a casual one, and Complex I may modulate cellular health through a different mechanism, probably by controlling the ratio of reducing equivalents.

In summary, our studies suggest that in primary hepatocytes arsenite dose-response relationship, with respect to growth and survival endpoints, does not follow either the threshold or the linear characteristics but rather a U-shaped model. Further, in our model system, although the mediator of arsenite-induced biphasic response appears to be ROS, the source of such ROS appears to result from nonlinear mitochondrial bioenergetics. Evidence for As-induced hormesis has been observed with respect to three distinct measures: cell proliferation or viability, base excision DNA repair, and, recently, telomerase activity [24, 47, 48]. Our results presented in this manuscript provide a fourth distinct measure of As-induced hormesis, mitochondrial bioenergetics, and suggest that mild stress can trigger a hormetic response mediated by the mitochondria that provide both short term metabolic benefits and the potential for long term benefits in increased stress resistance and longevity. Future studies should provide an opportunity to use this primary hepatocyte model to explore the signaling mechanisms that regulate the nonlinear effects of ROS and how cells respond to these signals that lead to either beneficial or harmful consequences.

## Supplementary Material

Arsenic exposure did not affect cellular ATP levels significantly. suggesting that the increased oxygen consumption in response to low dose arsenite exposure was not a result of increased coupling efficiency. Interestingly, when oligomycin was added to inhibit oxidative phosphorylation to measure coupling efficneicny, primary hepatocytes demonstrated increased leak respiration suggesting increased uncoupling of respiration.
