# Supplementary figures and images for: Arsenite Effects on Mitochondrial Bioenergetics in Human and Mouse Primary Hepatocytes Follow a Nonlinear Dose Response
Source: Oxid Med Cell Longev. 2017 Jan 9;2017:9251303. doi: 10.1155/2017/9251303 (PMC5253485; doi:10.1155/2017/9251303)

# SUPPLEMENTARY FIGURE 1. Arsenite effect on ATP levels in primary hepatocytes

A

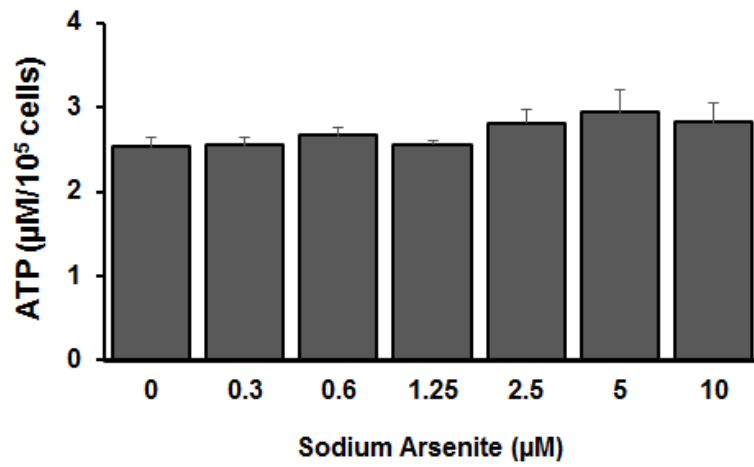

B

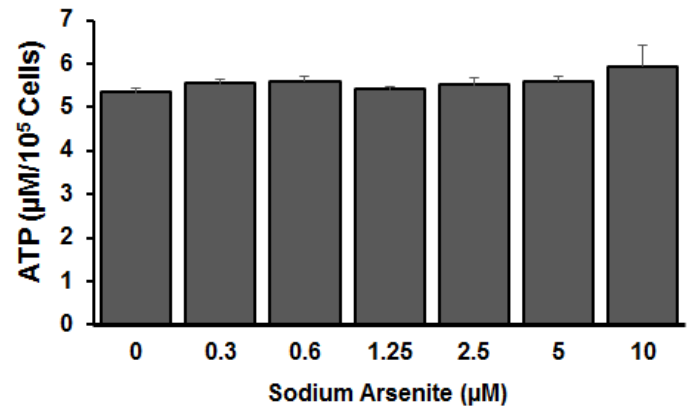

C

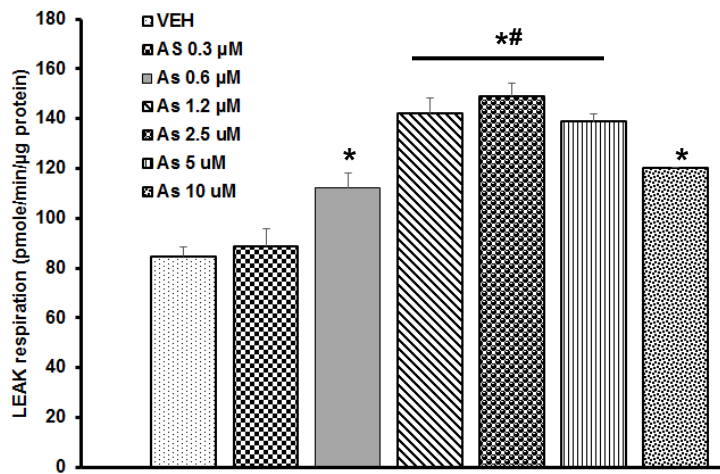

D

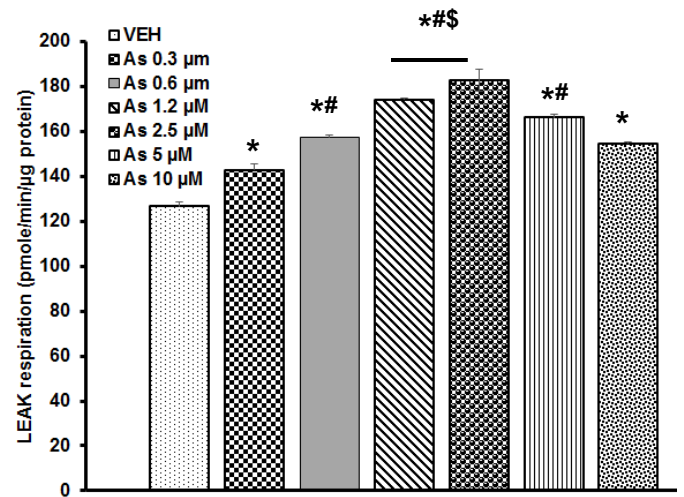

Supplement: Supplementary file 1 — Arsenic exposure did not affect cellular ATP levels significantly. suggesting that the increased oxygen consumption in response to low dose arsenite exposure was not a result of increased coupling efficiency. Interestingly, when oligomycin was added to inhibit oxidative phosphorylation to measure coupling efficneicny, primary hepatocytes demonstrated increased leak respiration suggesting increased uncoupling of respiration. [file 9251303.f1.pdf]
